# Factors That “Nudge People towards the Healthier” Snacks—A Qualitative Study with Student, Faculty, and Staff Leaders and Decision Makers

**DOI:** 10.3390/ijerph192315922

**Published:** 2022-11-29

**Authors:** Christie Leanne Kirchoff, Rumi Agarwal, Mariana Sanchez, Cristina Palacios

**Affiliations:** 1 Health Promotion and Disease Prevention, Florida International University, Miami, FL 33199, USA; 2 FIU Embrace, Florida International University, Miami, FL 33199, USA; 3 Dietetics and Nutrition, Florida International University, Miami, FL 33199, USA

**Keywords:** snack environment, food environment, college campus, healthy, barrier, facilitator, food policy

## Abstract

(1) College campuses pose numerous public health challenges for students, faculty and staff. The healthfulness of the snacks available on campuses is lacking, and there is a desire for change among the students and staff. The objective of this study is to understand the perspectives of the students, staff, and decision makers regarding the college campus food environment and the perceived facilitators and barriers to improving it. (2) In-depth interviews were conducted (*n* = 15) with decision makers in food, policy development, wellness, and nutrition at a large Hispanic-Serving University in South Florida. (3) The key stakeholders shared that educational campaigns, student buy-in, raising awareness around obesity and chronic disease, and the university’s position within the community would all help to facilitate improvements to the snack food environment. However, the participants noted that the complex nature of what is considered to be healthy and what divergent consumers want are significant barriers to improving the snack food environment along with concerns over lost revenue and the corporate structure. (4) These results inform potential focal points for multi-level interventions and inform policy discussions focused on improving the snack food environment at minority-serving universities. Taking strategic actions to improve the snack food environment may aid the students and staff of the university to enhance their diet quality.

## 1. Introduction

As obesity rates and the non-communicable diseases (NCD) associated with them continue to climb, the focus on how to curb these trends has shifted to identifying the contributors beyond the individual [1,2]. Identifying innovative ways to improve diet quality en masse is essential to curb the current NCD trends. For many years, obesity interventions focused primarily on the individual, and they did not serve to successfully curb the trends [3]. The new research suggests that there is a myriad of influences, contributors, and circumstances that create the epidemic of overweight and obesity and high adiposity which many countries are currently experiencing [4,5,6,7]. 

The research related to food environments is among the most promising new areas that has shed light on “the physical presence of food that affects a person’s diet” [8]. Food environments include neighborhoods, workplaces, and schools. The research examines the availability and accessibility of foods, as well as the political and socio-cultural impacts and influences on individual food choices [9,10,11,12,13,14]. Food environment research serves to lay the groundwork for policy and environment-level interventions to improve diet by auditing and grading the existing food environments. Utilizing tools to evaluate the healthfulness, marketing, availability, accessibility, and price of the foods available for purchase in a given location is informative for making intervention strategies that achieve the intended results [15,16,17,18]. A next step that supports the development of an effective policy is to convey the evidence-based research to the decision makers and to also gain an understanding of the barriers and facilitators that they perceive when one is thinking about improving the food environment. 

Obesity and overweight have multifactorial etiologies and multiple levels of influence. The ecological framework helps to explain some of the higher-level influences on dietary choices [19,20]. The framework consists of the macro-level and physical environments and the societal and individual factors [21,22]. The individual elements include knowledge, attitudes, beliefs, practices, demographic characteristics, preferences, motivation, self-efficacy, values, skills, and lifestyle. The social factors can implicate family, friends, and peers. The physical environment is associated with the places/spaces where people spend a large portion of time, and this includes workplaces, schools, homes, and neighborhoods. The physical food environment includes material elements that exert their influence through their presence in the field: the number and type of convenience stores (CS), vending machines (VM), restaurants, and products available in those spaces. Finally, the macro-level effects are the societal norms, industry standards, marketing, and governmental and institutional policies that influence food supplies and product trends [20]. 

College campus food environments are ideal sites for obesity and overweight interventions. The food environment is generally characterized as poor, and it serves as the primary food environment for emerging adult students who are newly learning to feed themselves as well as the local workforce (staff and faculty) employed at colleges and universities [9,10,11,12,13,14]. Recent trends in increased snack consumption and calories eaten outside of the home [15] make the snack foods available on-site at a commuter school particularly important. Snack foods in particular are of concern due to the nature of the student population at Florida International University (FIU), where greater than half of the students identify as food insecure, more than 15,000 of them attend weekly classes after other food venues are closed, and approximately 3000 (<1% of the student body) of them live in on-campus housing. Commuter schools educate student bodies that are largely commuting to school rather than living on campus, and frequently, they work in addition to attending college compared to other universities that typically house greater than 22% of their student body on campus. There exists a substantial body of research detailing both the notoriously poor food environments and the susceptibility of college students to poor nutrition choices [9,10,11,12,13,14]. Examining these factors within minority-serving institutions is of notable significance given the well-documented obesity-related disparities experienced among Hispanic and non-Hispanic Black adults [15]. What is less well understood is how the key stakeholders in college campuses perceive the snack food environment and the barriers and facilitators to improving it. Therefore, the aim of this exploratory qualitative study is to identify the themes regarding the barriers and facilitators to the provision of healthy snack foods across the ecological levels of influence at a minority-serving institution. This study was part of a larger study that examined the snack food environment at the university and the faculty, student, and staff perceptions of the campus environment. The results from this study will inform the development of intervention strategies to improve the snack environment and identify approaches that will facilitate decision maker buy-in.

## 2. Materials and Methods

This is an exploratory study of the barriers and facilitators to improving the snack food environment at a minority-serving institution. In-depth semi-structured interviews were conducted with the students, faculty and staff partners, and decision makers at FIU, and they focused on exploring perceptions of the snack environment and eliciting the potential ideas, barriers, and facilitators to improving it. 

The study was conducted at FIU, a multi-campus public research university in South Florida with a student body of nearly 56,000 students (spring 2021 enrollment) and 10,000 faculty and staff. The racial/ethnic composition of FIU’s student body is 61% Hispanic, 15% White Non-Hispanic, 13% Black, 4% Asian/Pacific Islander, and 7% other minority groups. An audit of the snack environment, all of the VMs and CSs on campus, was conducted at FIU, and consisted of 76 snack food VMs, 121 drink VMs, 8 ice cream VMs, and 2 CSs. This audit revealed that the CSs ranked in the least healthy range, and more than 84% of the snack foods available in the VMs were unhealthy (>200 calories, or >230 mg sodium, or >35% of calories from fat, etc.) utilizing the Nutrition Environment Measurement Scale for VMs [23], with the remaining 16% of the healthy items concentrated in 12 machines throughout the 342-acre campus [24]. In addition, the audit revealed that choice architecture, which is defined as the manipulation of the price, quantity, and/or display of the items to encourage the purchase of some items over others [5], was being utilized in both the VMs and CSs to promote the purchase of very unhealthy items. At the subject university, the dining hall and individual restaurants are managed through one contract, while the snack VMs were managed through a different contract, the beverage VMs were managed through another, and the ice cream VMs were managed through another, and the CSs were managed through two contracts, where the revenue generated from these contracts was used for employee incentives and bonuses, scholarships, and other miscellaneous expenditures. 

The ecological framework informed the purposive sampling of the participants such that representatives across each level of influence were sampled. A sample frame was generated by reviewing the FIU websites for the employees that held positions related to food purchasing, vendor selection, policy development, student and employee wellness, and food services. In addition, student organizations and faculty senate committees related to student government, nutrition, physical activity, and policy were also included. Once they had been identified, the participants were recruited via email to participate in recorded zoom interviews between October and December 2021. In total, 15 individuals completed an interview. All of the participants provided their written consent prior to participating in the interview. The study received ethical approval from the FIU Institutional Review Board (#110611).

One interviewer (C.L.K.) used a semi-structured interview guide to facilitate the interview. While the interview was structured to elicit perceptions of the snack food environment and food marketing strategies, the interview also permitted participants and the interviewer to deviate from the core questions and explore topics further. The semi-structured interview guide contained five sections. The first one was used to prompt responses that would give insight into how the individual stakeholder made decisions and viewed their role within the university. The second section aimed to elicit perceptions regarding the snack environment, definitions of what the snack food environment is, its healthfulness, and comments on the preliminary data collected from an audit of the VMs mentioned above. The remaining three sections were structured to encourage the separation of the barriers, facilitators, and ideas for improvement. The interviews were conducted via Zoom conferencing, they were audio-recorded, and they lasted between 60 and 80 min. A brief demographic questionnaire accompanied the interviews, and it included the length of employment, job title, gender, race/ethnicity, and the number of hours spent on campus.

### Data Analysis

Audio recordings of the interviews were transcribed verbatim, and they were checked for accuracy. An inductive and deductive coding strategy was used to code the transcripts. The inductive coding phase included the production of “open codes” by conducting line-by-line coding of the transcripts, which was followed by a second stage of “axial codes” that were applied to the open codes [25]. In total, 398 open codes and 30 axial codes were produced. The initial axial codes were combined to produce a final set of 20 axial codes. One axial code was removed for analysis elsewhere. To assess the reliability of the axial codes, a second coder (R.A.) applied a subset (80%) of the axial codes to a subset (25%) of the open codes which were chosen at random. The overall inter-coder agreement was 81%, and the Cohen’s Kappa was 0.83, which falls within a substantial range [26,27]. The divergence in the axial codes was discussed among the coders, and it was resolved. In addition, throughout the research process, reflections on the potential power dynamics at play in the participants’ responses and how to interpret them were discussed with the research team. Finally, the tertiary codes were developed from the axial codes to identify the broadest themes identified by the axial codes. Once the inductive process was completed, the ecological domains (individual, social, physical, and macro) were applied to the axial codes as a deductive coding step. The tertiary codes were used to structure the presentation of the results. Table 1 illustrates the axial codes and the alignment of the five inductive and deductive tertiary codes.

## 3. Results

The participants included two students, two faculty, and eleven staff members (Table 2). The results are organized into the following thematic areas: 1. Perspectives regarding the definition of snacks and a description of the snack food environment, 2. The participants’ perceptions of how and what influences the snack choice, 3. The ideas regarding how to improve the snack food environment, and 4. The perceived facilitators and barriers to changing the snack food environment.

### 3.1. Perspectives Regarding the Definition of Snacks and Description of the Snack Food Environment

The participants described what they believed constituted the snack food environment, and they defined the term snack. Most of the participants defined a snack as it related to the traditional North American pattern of consuming three meals (breakfast, lunch, and dinner) per day, either by the snack’s timing, size, or content compared to a meal, while other participants felt that multiple snacks consumed together could constitute a meal. An administrator defined a snack this way:

“So, I typically look at the time of day that you’re eating it. If it’s outside your normal breakfast, lunch, or dinner time, I would consider that a snack, as far as what it is I think it can be a variety of things for a variety of people.”

The participants shared that a snack could be any size or item that was eaten between meals or to tide one over until they ate a meal. Several participants felt that the size of the snacks helped set them apart from a meal, using descriptors such as small, portioned, and fewer calories. For example, one executive said, “I mean a snack, definitely means like smaller portion. And I think it also means like convenience and affordability like it’s the combination of that”. A few participants suggested that a snack could only be small items such as chips, crackers, cookies, or grapes, but it could not include beverages. The participants also shared individual components of what constituted a snack, and they included healthfulness and nutritional density definitions.

There was diversity in the participants’ descriptions of the snack food environment, with some of them including all of the food venues available on campus and others describing only the CSs and VMs. These descriptions were generally predicated upon how a snack was defined and what the content of a snack could be. For example, an administrator, who believed that snack foods can be a “variety of things to a variety of people”, defined the snack food environment to include “the convenience stores that are located on campus both the one and housing and the one in the graham center [main building on campus with food and snack outlets, social spaces], the dining facilities that students may choose to use as, as their source for snacks”. The participants who defined a snack as any food tended to describe the snack environment as inclusive of all of the venues, whereas those who had specific food items in mind (chips, cookies, and candy bars) when they were defining a snack only included the VMs and CSs.

### 3.2. Decision Makers’ Perceptions of How and What Influences Snack Choice

The factors that affect snack choices were discussed by the participants throughout the interview, and they included the individual, social, physical, and macro-level influences. Many participants mentioned that an individual’s demographic characteristics, such as age, gender, culture, and socioeconomic status, impact their snack choices, encompassing both the individual and social levels of influence. For example, a faculty leader shared:

“Well, you have to look at the general attitude towards food externally, and what goes on and there’s a cultural perspective I always think that, you know, I do not know what Hispanic families, eat, non-Hispanic family members or African Americans so it starts with what is accessible what your parents buy when if you’re not in charge of buying the groceries in the house and how much money the parents have to spend and then what is it going to buy the fresh, frozen green beans or are they going to get the green beans in the can.”

Another individual-level influence that was mentioned frequently was the fact that the students on campus after a particular time of day would be forced to use the VM or find an open food vendor. With the vast majority of restaurants and CSs closing between 6 and 7 p.m., the participants described how this limited the range of choices available to only the VMs. A student leader put it this way:

“The problem is when we as students spend eight hours in the library after 8 p.m. to 6 a.m. There are no on-campus vendors at the time so you’re eating Pop-Tarts for meals. You might need a pop tart in a bag of chips, as your late-night meal. When that is not what most would consider a meal.” 

Some of the participants perceived the campus snack food environment as being “balanced” (equal parts healthy and unhealthy). In contrast, others felt it was imbalanced (more unhealthy than healthy), and others felt that healthy options were available for people who were willing to look for them. One wellness and policy executive described the food environment this way: “So if they want the healthier option they can go find it, because it exists in X Y or Z ….”, while another executive described it this way: “I think for faculty and staff that work here you know I think there are not that many alternative options that are health-wise that are quick to grab”. From the student perspective, one student leader said, “I’m not surprised by them [proportion of unhealthy to healthy items on campus] I think it matched what I said. We do have vending machines that offer healthy options but they’re not everywhere”, and another described it this way: “I do think that there is an imbalance because you can go somewhere and there are not healthy options um not as nutritious, nutritious options”.

The participants identified two macro-level influences of snack choice: affordability and choice architecture. The participants expressed that they felt that healthy products were more expensive than unhealthy products were and that consumers would make decisions based upon a calculation of what would fill them up for the least amount of money. For example, one wellness executive said: “I think it’s crucial that if somebody only has $1 or 2 to spend that they’re spending it wisely. And that’s going to be, you know so we need to make sure that you have healthy snacks within that price point”. Another wellness executive explained how “unfortunately, they have to make those hard decisions right like yeah I want a banana, and I know bananas are cheap but like right like I want a banana but you know like cookies like 50 cents less and I could use those 50 cents for gas”. 

Marketing in the form of product placement, the quantity of items, and the pricing were other emergent themes related to the macro-level influence on snack choice. Many stakeholders described that they understood that the choice architecture influenced the snacks that were purchased, as the buyers favor the purchase of unhealthy foods, with one faculty member stating: “you know what, we know that these are the factors that enhance the marketing of unhealthy food and we do not want those factors we want the opposite we want the eye level to be something that’s a little bit more nutritious”. A student leader suggested that student preferences influenced how VMs and CSs were stocked and the items that were displayed, saying:

“But if the students weren’t going to buy it, it wouldn’t be in that area, we’re not putting the granola bar in the area of prominence. If students aren’t buying the granola bar. I think that the choices that these on-campus vendors are making relate to items that they believe or have research on that are highly mobile. They put a box of chocolate bars out the box of chocolate bars goes in an hour. So, you put more chocolate bars. Right, that’s the architecture, you get a higher turnaround on them.” 

### 3.3. Ideas Regarding How to Improve the Snack Food Environment

The decision makers expressed their ideas about how to improve the snack environment through a range of potential direct and indirect actions. The most commonly described indirect action was the education of consumers about personal nutrition and health. Many participants shared that a campaign either within a mandatory first-year course or throughout the campus on all of the retail food outlets would improve the choices that individuals make when they are purchasing all food types. One student leader suggested:

“We do have areas where we can teach our students. Every student is required to take an SLS [student life skills] intro to FIU course. I think that working with the Office of the Provost to add a module about nutritional information would be useful. We have modules about leadership modules about University activity modules about financial wellness. So, I think that your actual wellness should play a role in that as well, especially at a university.”

Similarly, indirect and at the individual and macro levels of influence, the stakeholders suggested that the research be used to identify what consumers would purchase, rather than letting the purveyor dictate what is available. A student leader stated, “I also think maybe if we did implement it (increasing the number of healthy items in each VM) and that surveying students and finding what kinds of healthy portable snacks that would be fine in a vending machine, finding out what they would actually eat”.

At the social level, the participants voiced that having a leader or champion and incorporating the student’s voice were necessary to effect change. Many participants believed that change was not possible without a single unifying leader who would engage collaborators and partners to move things forward.

The direct actions that were suggested to improve the snack environment included those at the physical and macro-level which were focused on changing the available snack options and developing policies to address the nutritional quality of foods served throughout the entire campus. Most of the participants that suggested changing the availability of the snack options described how the ratio of healthy to unhealthy offerings should be shifted towards healthier options. An administrator explained it this way:

“I think when we look at the snack machines having a more balanced representation of healthy snacks versus snacks that’re high in calories and high in fat would be very important. And I think that they would be surprised about how much of that would actually be purchased. If it was available.”

Many others suggested that choice architecture be utilized to nudge consumers towards making healthy choices. For example, one wellness executive stated, “I do think that some type of choice architecture should be utilized, but it should be utilized to nudge people towards the healthier, and not towards the unhealthy, or the one that’ll fly off the shelves”. Still, others recommended increasing the number of dedicated “Healthy” VMs and reducing the prices of healthy options. The policy discussions centered around policy as a means to address behavioral outcomes, including the implications, concerns, and reservations about doing so. For example, an administrator described policy this way:

“Well, you know, when it’s a health-related thing that’s hard because I don’t generally like a mandate kind of thing. But when it’s for the greater good, which I think healthy food definitely is. Then, you know, like a tobacco policy or an alcohol policy, things like that are put into place, to encourage healthy behavior. I’m much more supportive of than mandates around, I guess, others. I mean, I wouldn’t even know how to categorize like other social behaviors. It’s weird because healthy eating and nutrition. At the end of the day, it’s a very personal choice. But then, so too is smoking. And if somebody is obese, that doesn’t impact my health in the way that them smoking next to me does. So, I think it’s a fine line. I don’t know, I mean I applaud the places that are doing it because they value the health of their community. But I don’t know, I don’t know how it would be received someplace like here.” 

### 3.4. Facilitators of Changing the Snack Food Environment

Multi-level levers of change were discussed in a broad sense regarding the snack food environment. There was a crossover between education/training as a method of facilitating change and incorporating the students’ voice, which were ideas to improve the snack food environment and facilitate the uptake of any changes to it. Many people shared that without an educational marketing campaign to accompany the changes, those changes would be ineffective at improving the individuals’ choices. Several others emphasized that implementing the changes made with input from the students would be more widely accepted than a top-down approach would be. Incorporating the students’ voice was described by an administrator as follows:

“So, I think the key is making sure that you know when we’re looking at. You know, how we increase the number of healthy snacks available. That we’re talking to the students and finding out, you know, what are some things that are popular with them. Because if we’re doing that, the odds are that the decrease in revenue is going to be minimal. But if we’re as administrators trying to figure out what that is, we are gonna miss.”

The decision makers often framed these suggestions in the broader context of the university’s role within the larger (non-university) community, expressing a duty to care for the students which were entrusted to them and others embracing the broader role of a university to its community. For example, an executive described efforts to persuade other higher-ups like this:

“I said yeah, you know, you’re training and getting people graduated who would not otherwise have a shot, and it’s extraordinary and you’re giving them socioeconomic lift and- but guess what? They’re still dying 10 years before everybody else, because they’re not as healthy. So, we got to do both things, we must get the health literacy up of the county. And part of that, like I said movement, you know, nutrition, is there, so maybe that’s, you know, we shouldn’t be so insular in this work, and maybe there’s a win for the county. You know the county has lots of places where we can have influence and the kind of snacks that are available. If they come along with us, then that’s a better, that’s a different story.” 

Another macro-level influence that the participants discussed was the idea of an awareness of an issue and how far up that awareness travels within an organization’s structure. The stakeholders related stories of alcohol and tobacco initiatives that first required the decision makers to gain an understanding of the problem, suggesting that the rise in obesity rates may prompt this level of awareness and facilitate change. One executive explained that:

“I know that there is a lot of data to like the smoking prevention and things like that that could be quite beneficial, especially when you look at the addiction or the type of addiction type component to like the added sugars… The content of the food things there. So, I think there could be some benefit that would be able to utilize that type of model.” 

At the same time, the others believed that obesity was nowhere on the radar of the university leadership.

### 3.5. Barriers to Changing the Snack Food Environment

Three of the five barriers to change identified by the stakeholders could be classified as macro-level influences. The university’s organizational structure was the most frequently cited barrier to improving the snack environment. The idea that decision makers are being pulled in many different directions which affects both the prioritization and awareness of the issues was discussed at length. A wellness executive explained this difficulty as follows:

“Another hard issue is for these departments. They have to meet different constituent needs, right? So, they have the student constituent, they have the employee constituent, they have visitors. You know, there’s always a sense of compromise and like, meeting in the middle. So, you lose out on some of those prioritizations, right? So, yeah if you might be talking about student healthy eating habits. You know okay well does the employee voice matter less, or matter more, or equally?” 

Furthermore, the structure of the state university system was described as being more dependent on revenue generation and less dependent on the healthfulness of available snack food options. An administrator perceived it as follows:

“I think the financial piece is probably the biggest. Yeah, you know, because I know that the vending machines make the university a lot of money. And, you know, to the point that you made earlier, those dollars that come in from those things do support a lot of important programs. So, I think that that’s probably the biggest barrier like people don’t want to rock the boat, because that’s a secure sense of, you know, source of income…. I definitely think the money is the biggest obstacle.” 

The participants viewed other facets of the university’s structure as presenting significant barriers, including difficulty with communication between the departments and a hierarchical, top-down structure that is inherent to the university’s everyday operations. One executive described these barriers in this way: “there’s a lot of silos that want to stay siloed, and may not want to collaborate. So it kind of makes it a little harder if you know someone doesn’t want to come play at the playground with you and get all that messaging out all together”, and another executive stated, “but it does require, you know, institutional will and that is top down right so I have to convince the president the Board of Governors not just me, we all have to think that this is important work for us”.

From a social perspective, many stakeholders described how the diversity of the consumers and stakeholders by age, culture, amount of time on campus, and priorities makes it nearly impossible to please everyone or address every problem. On an individual level, the decision makers generally perceived that there was low collective knowledge and consensus within the university community regarding what constitutes “healthy” eating. The participants described the term “healthy” as forever changing and subjective, making it difficult to improve the snack environment and create educational campaigns. An executive explains their interpretation of healthy like this:

“Healthy is a dangerous word, means different things to different people…. do not think consumers (those surveyed) know what healthy means. Branding plays a role in the perception of healthy and does not necessarily reflect salt, sugar content, not necessarily healthy.”

## 4. Discussion

The purpose of this study was to understand the key informants’ perceptions of the snack food environment and the barriers and facilitators to improving it. The results contribute added depth and context surrounding four distinct themes: varying definitions of what constitutes snack food and the snack food environment, the range of perceptions regarding the factors that affect snack choice, suggestions for improving the snack environment, and the identification of the local barriers and facilitators to improving the snack food environment. Within each thematic area, the participants’ responses indicated many levels of influence which are posited by the ecological framework, reemphasizing the multifactorial nature of food choice. This qualitative analysis also revealed contingencies between the ecological levels of influence, which informs an understanding of the complexity of how multi-level dynamics are implicated in college campus food environments.

First, it is notable that there were variable conceptualizations of what constitutes a snack and the snack food environment. While participants were generally comfortable sharing their perspectives on these two items, they were quick to suggest that others may not agree with their perceptions, suggesting that the definition of a snack/snack food environment was a matter of personal opinion. Snacking practices have changed radically in the past 20 years, with the number and types of prepackaged ready-to-eat foods increasing dramatically during that time [3,17,28], which might help to explain why definitions of snacks and snack food environments differed or were perceived as being opinions. Many of the participants defined snacks in relation to meals, differentiating snacks based on size or timing compared to meals, while others defined snacks as certain items or in relation to their needs. These results are similar to those of Younginer et al. (2016), where the low-income caregivers’ definitions of snacks for their children fell into five domains that were similar to the current findings: types of food, portion size, time, location, and purpose [29]. In their research, the domains of types of food, portion size, and time were most closely aligned with how the stakeholders defined snacks in the current study. The themes of portion size and time were also most frequently discussed in relation to meals [29]. It is finally worth noting that the varying definitions of snack and the snack food environment identified in this current study may reflect broader epistemic uncertainties regarding what constitutes a snack as the researchers also hold differing definitions of snacks [18]. The current research helps to illustrate how a clearer definition of snacks will improve the quality of research in the future. Defining snacks more objectively will allow researchers to compare findings across locations and samples, identify specific intervention points, and examine areas in need of more detailed research.

The key informants expressed that snack choice was influenced by demographic characteristics, affordability, accessibility, and the physical environment. There exists a body of research detailing the influences on snacks and food choices which suggest that demographic characteristics, affordability, and accessibility influence consumers’ choices [29,30,31,32]. However, to our knowledge, this is the first study in which the decision makers in a university environment have been queried on the topic. It is important to understand from the decision maker’s point of view what they believe influences the choice as these beliefs may modify the implementation of interventions that target this choice. 

That the current study revealed that the decision makers understand that the physical environment influences food choice, is significant. The physical environment’s influences on snack choice which were discussed by the participants included the choice architecture and after-hours choices. The decision makers expressed that the students on campus after a particular time of day would be forced to use the VMs or find an open food vendor. This may reinforce unhealthy eating in ways that supersede knowledge and other modifiable factors for health interventions, which drives home the importance of paying attention to the physical environment [30,33,34,35,36]. This may help to explain the differing results seen in various research studies related to the individual-level interventions to improve snack choices, such as increasing the number and labeling of healthy options in a limited number of machines, and pricing strategies, where some research suggests that these interventions work, whereas others have found no effect without an understanding of the overall food environment and availability of other snack options. 

Furthermore, the discussions around choice architecture reveal that the participants are aware that it has an impact on choice whether they agreed ethically with its use or not, although those participants who felt that the vendor had a right to use it held more decision-making power than those who felt the university should decide or have a say. Interestingly, choice architecture and the nudge theory have been explored as ways to improve food environments with far-reaching impacts [4,5,37]. For example, Walmsey et al. 2018 reported increased fruit and vegetable sales when they used choice architecture to increase fruit and vegetable purchasing behaviors by increasing the prominence of fruits and vegetables within a university campus grocery store.

The participants’ suggestions for improving the snack food environment included educating the consumers, conducting research to identify the needs, soliciting student input, identifying a leader, improving the content of the VMs and CSs, and developing a policy to address the proportions of healthy versus unhealthy offerings. These concepts are partially in line with the results from research among students at FIU in 2018 and 2019, which indicated that they too felt that the content of the VMs should be improved and that educating and enticing consumers to use healthy VMs was important [32,38]. These ideas are also in line with the strategies that other universities, municipalities, and researchers have used to improve their snack food environments [39,40,41,42]. Both the University of Michigan (UM) and the University of California (UC) have developed policies related to the snack food environment that included increasing the proportion of healthy items offered and identifying and enrolling key stakeholders to lead the efforts. The UM has also incorporated a labeling/education component to make it easier for students to identify healthy products, whereas the UC piloted healthy VMs prior to implementation.

Perceptions of facilitators included education, student input, the role of the university, and whether the topic of concern is on the radar of university leadership. As mentioned earlier, education and student input have both been used to improve individual behavior. In the current study, the discussion deepened to using education and student input to complement a multi-pronged or policy approach to improving the snack food environment. Universities and municipalities with snack policies have incorporated a nutrition education/marketing aspect to their policies that helps the consumers to identify the healthy options [39,41,42]. While this is often categorized as education, it differs from what the participants in the current study seemed to be suggesting, which was an actual education of the consumers to empower them to be better caretakers of themselves. Perhaps this nuance is related to the fact that the participants were asked for their perceptions of the barriers and facilitators prior to any action or discussion on the topic. It is possible that once policy discussions and actual changes take place, this component would become its own policy rather than a part of a snack food policy.

With regard to the university’s role in caring for the student and its workforce, the participants’ responses ranged from utilitarian concepts of intervening in the best interest of the majority to a corporate responsibility concept that positioned the university within its community as a role model and brand. The participants described a moral imperative to act if it is known that obesity and NCDs are linked to the food environments. During these discussions, the participants frequently mentioned the impetus to act in the case of alcohol and smoking on campus as a protective measure but also as a message to the broader community that these behaviors are unhealthy.

The barriers to changing the snack food environment included university structure, the divergent needs of the students and stakeholders, revenue, and a lack of consensus on the meaning of “healthy”. The structural components of the university that were described as being the barriers to change included the organizational structure, communication, priorities, and existing policies and buy-in from governing bodies. Adding a layer of complexity was the hierarchical nature of a state-funded university and the role of independent revenue generation to meet operational needs. The loss of revenue, therefore, was also implicated as a major barrier to change. While organizational structure has been found to be a barrier in other research [6,43,44], the complicated dynamics of the state university system have not been mentioned in this detail prior to the current study. So too, the loss of revenue has been found to be a barrier to implementing and designing policies to improve food environments in prior research [6,7,43,44]. Other universities have utilized pilot studies to identify the foods that consumers will purchase to offset this concern, while others have incorporated substantial marketing campaigns to do so [39,40,41]. However, the participants in this study discussed how revenue from VMs which is used for bonuses, scholarships, and recruitment incentives were implicated when they were considering making changes to the snack food environment.

Similar to the discussions around the definition of snacks, there was consensus that what is considered to be “healthy” is not static. The idea that the participants felt that what is “healthy” was a moving target and rife with complexities, could partially be explained by the relative novelty of nutrition science especially when it is translated to the public. However, those that expressed that the term “healthy” was ever-changing or “dangerous” tended to be higher up the decision-making ladder compared to those who simply felt that others may disagree with their definition. It is interesting to note that all of the discussions of what is healthy evolved spontaneously from the word healthy being used, whereas the participants were asked to define snacks and the snack food environment, specifically. 

Stakeholder and consumer diversity was seen as a barrier to improving the snack food environment, with the participants suggesting that it is impossible to meet all of the consumers’ nutritional needs and for them to participate in every colleague’s campaign. This too is a new finding that may be attributable to the timing of this research compared to other post-policy research.

Taken together, the findings of this research highlight the multifaceted influences on the food environments and food choices that can inform tailored interventions, improvements to the physical environment, and avenues for research. The fact that the participants are aware of many of the main influences for which they are responsible, coupled with many participants’ belief that action should be taken to improve the snack food environment suggests that buy-in may not be that far off. Interventions aimed at improving public health knowledge and nutrition knowledge could aid in improving individual and social level behaviors and perceptions. The physical environment can be improved through interventions to ensure that healthy options are available in all VMs and CSs at all times, altering the VMs and CSs to reflect a larger proportion of healthy rather than unhealthy snacks, healthy item identification within VMs and CSs, and pricing strategies. Macro-level interventions could include creating a sustained institutional memory in campus institutions that manage snack food services and designating a food environment committee. Future research should be geared towards improving snack definitions, piloting nudge interventions, which use marketing and placement to make the healthy choice the easier choice, identifying healthy items that consumers will purchase, and understanding the stakeholders’ perceptions of implementing policies that are aimed at changing behaviors.

### Limitations

This study had several limitations. Firstly, the qualitative findings are not generalizable to other universities due to the small sample size and in-depth attention that was paid to the participants’ perspectives in this context. However, the findings may be similar at other universities with comparable organizational structures and student bodies. Secondly, the sampling strategy aimed to enlist a broad range of participants from multiple departments within the university, which may have eschewed an elicitation of divergent viewpoints within departments had the entire department been sampled instead. Lastly, the COVID-19 pandemic may have had an impact on this research with regard to willingness to participate, and this could have affected perceptions of health, barriers, and facilitators, given the drastic changes in everyday activities and operations of the university. 

## 5. Conclusions

In this qualitative study of student, staff, and faculty leaders and decision makers at a minority-serving institution in South Florida, the interviewees described food choice as being impacted by several factors at multiple levels. The participants shared perceptions and definitions that evinced all of the levels of the ecological framework, ranging from the individual-level influences on food choice to the macro-level influences on policies that impact food availability. These results inform potential focal points for multi-level interventions, and they also inform policy discussions focused on improving the snack food environment at minority-serving universities. Taking action to improve the snack food environment may aid the students and staff of the university to improve their diet quality, and may send a clear message to the community that the university cares about providing a healthy environment. Furthermore, the findings from this research can help to improve future research by establishing clear definitions of words and phrases that are integral to the study of snack food environments. 

## Figures and Tables

**Table 1:** Axial and tertiary codes and descriptions.

Tertiary Code	Axial Code	Description	Ecological Level of Influence
Snack	snackdef	Personal definition of what constitutes a snack.	
	Snackenvirodef	A personal description of what is the snack food environment.	
Choice	demographics	Eating patterns are influenced by gender, gender norms, gender within cultures, age, and SES.	Individual/Social
	afterhours	Snacking options change after the restaurants and stores close and where you are on campus.	Physical/Individual
	enviroperc	Perceptions of the overall snack food environment’s healthfulness based upon data provided and/or personal observation.	Physical
	afford	Snack choice is influenced by price and affordability, and snack prices differ between healthy and unhealthy options.	Macro
	choicarc	Perceptions of the role of choice architecture in snack choice, and its place in business and university settings.	Macro
Idea	education	Using nutrition education/marketing and identifying healthy options as a means to both improve the snack environment and facilitate the uptake of any changes.	Individual/Macro/Physical
	research	More research is needed in order to address the issue of improving the snack food environment and other university-wide initiatives.	Macro/Individual
	leader	A champion, leader, or committee needs to lead the charge to change the snack environment.	Social
	studvoice	The student voice can and should be incorporated into all aspects of planning, implementation, and buy-in related to altering the snack environment, including how to best reach them.	Social
	improvecontent	Increasing the number of healthy options to improve the overall snack food environment.	Physical
	policy	Discussion of policy as a means to address behavioral outcomes, including, implications, concerns, similarities, and reservations.	Macro
Facilitator	education	Using nutrition education/marketing and identifying healthy options as a means to both improve the snack environment and facilitate the uptake of any changes.	Individual/Macro/Physical
	studvoice	The student voice can and should be incorporated into all aspects of planning, implementation, and buy-in related to altering the snack environment, including how to best reach them.	Social
	pillar	The university should take action because of its unique position within the community as a leader and pilar which is responsible for helping to form students into adults.	Macro
	radar	An opinion, experience of inquiry, or discussion related to the investigation into food environment, snacks, nutrition, obesity, and students’ eating on campus.	Macro
Barriers	whatishealthy	The concept of what is healthy varies and changes from person to person and over time, and trends and what one wants to eat also change over time, so to include the idea of what is healthy is difficult.	Individual
	stkdiverg	The fact that stakeholders and consumers are so diverse in age, culture, time on campus, priorities, and needs means that meeting all their needs will be a challenge.	Social
	radar	An opinion, experience of inquiry, or discussion related to the investigation into food environment, snacks, nutrition, obesity, and students’ eating on campus.	Macro
	revenue	Changing the food environment’s impact on revenue is a barrier to it.	Macro
	structure	The university’s business structure (organizational, communication, priorities, existing policies) and buy-in from higher-ups are major influences on improving the snack environment.	Macro

**Table 2:** Demographic characteristics of interviewees, *n* = 15.

	Male (*n* = 6)	Female (*n* = 9)
Position		
Executive	2	2
Wellness	1	2
Policy	0	1
Student/Faculty	2	2
Food Services	0	1
Administrator	1	1
Years at FIU		
1–5	1	1
6–10	3	3
>10	2	5
Race/Ethnicity		
White	3	3
Hispanic/Latino	2	6
Middle Eastern	1	0

## Data Availability

The data presented in this study are available on request from the corresponding author. The data are not publicly available due to privacy restrictions.
